# Ursolic acid prevents doxorubicin‐induced cardiac toxicity in mice through eNOS activation and inhibition of eNOS uncoupling

**DOI:** 10.1111/jcmm.14130

**Published:** 2019-01-04

**Authors:** Haiman Mu, Haiwen Liu, Jiayi Zhang, Jianhua Huang, Chen Zhu, Yue Lu, Yueping Shi, Yi Wang

**Affiliations:** ^1^ First Affiliated Hospital of Jinzhou Medical University Jinzhou China; ^2^ Graduated School of Jinzhou Medical University Jinzhou China; ^3^ Life Science Institute of Jinzhou Medical University Jinzhou China

**Keywords:** apoptosis, cardiac toxicity, doxorubicin, ursolic acid

## Abstract

In addition to the known antitumour effects of ursolic acid (UA), increasing evidence indicates that this molecule plays a role in cardiac protection. In this study, the effects of ursolic acid on the heart in mice treated with doxorubicin (DOX) were assessed. The results showed that ursolic acid improved left ventrical fractional shortening (LVFS) and left ventrical ejection fraction (LVEF) of the heart, increased nitrogen oxide (NO) levels, inhibited reactive oxygen species (ROS) production and decreased cardiac apoptosis in mice treated with doxorubicin. Mechanistically, ursolic acid increased AKT and endothelial nitric‐oxide synthase (eNOS) phosphorylation levels, and enhanced eNOS expression, while inhibiting doxorubicin induced eNOS uncoupling through NADPH oxidase 4 (NOX4) down‐regulation. These effects of ursolic acid resulted in heart protection from doxorubicin‐induced injury. Therefore, ursolic acid may be considered a potential therapeutic agent for doxorubicin‐associated cardiac toxicity in clinical practice.

## 1 INTRODUCTION

Doxorubicin is an anthracycline antibiotic that is commonly used for the treatment of tumours, such as lymphoma, leukaemia and breast, ovarian, lung, gastric and thyroid cancers, in clinical practice. However, it has severe side effects on the heart, leading to cardiomyopathy and/or congestive heart failure in cancer patients.^1^ ROS are among the major factors that damage cardiac cells after doxorubicin treatment.^2^, ^3^ Thus, synthetic agents such as antioxidants and metal chelators have been used to prevent doxorubicin‐induced cardiac toxicity.^3^, ^4^ Nevertheless, the clinical effectiveness of antioxidant therapies remains poor. Recently, herbal and natural products with antioxidant capacity have been assessed for the prevention of doxorubicin‐associated cardiac toxicity.^5^, ^6^, ^7^, ^8^, ^9^ Because many herbal and natural products such as ginsenoside Rg1 are safe with lower human toxicity, such products that can prevent cardiac toxicity after doxorubicin administration are highly desirable in clinical practice.

Triterpenoids, the main constituents of multiple traditional medicinal plants, have been assessed for their antitumour, liver protective, analgesic, anti‐inflammatory and cardiac protective properties. Among triterpenoids, ursolic acid is found in many medicinal plants, including *Melaleuca leucadendron*,* Eriobotrya japonica*,* Ocimum sanctum*,* Rosmarinus officinalis* and *Glechoma hederacea*.^10^ Ursolic acid is found as a free acid or aglycone for triterpenoid saponins. It is widely known for its antitumour effects,^11^, ^12^, ^13^ and possesses strong hepatoprotective activity against ethanol.^14^ Balanehru et al^15^ reported that the protective effect of ursolic acid against free radical damage is apparently stronger in the heart compared with the liver under in vitro conditions. It was further demonstrated that ursolic acid protects the heart from oxidative stress in vivo.^16^ However, few reports have evaluated the effects of ursolic acid on doxorubicin‐induced cardiac toxicity. Because ursolic acid enhances the anticancer effects of chemotherapy,^17^, ^18^, ^19^, ^20^ it would be of great clinical significance if ursolic acid protects the heart from doxorubicin‐associated toxicity.

This study aimed to assess whether ursolic acid prevents doxorubicin‐induced cardiac toxicity. The results showed that ursolic acid prevents cardiac toxicity after doxorubicin treatment by increasing the phosphorylation levels of AKT and eNOS, and inhibiting eNOS uncoupling through NOX4 down‐regulation. These findings suggested that ursolic acid may be considered clinically for cardiac protection in the context of doxorubicin administration.

## 2 MATERIALS AND METHODS

### 2.1 Reagents

Doxorubicin was purchased from Aladdin Industrial Company (Shanghai, China, purity 98%). Ursolic acid was obtained from the National Institutes for Food and Drug Control (Beijing, China). Anti‐p‐Akt (Ser473), anti‐AKT, anti‐p‐eNOS (Ser1177), anti‐eNOS and anti‐cleaved caspase‐3 antibodies were from Cell Signaling Technology (Shanghai, China). Anti‐NOX4 was purchased from Novus Biologicals (Shanghai, China).

### 2.2 Animals

All animal experiments were approved by the Experimental Animal Ethics Committee of Jinzhou Medical University, and conformed to the Guide for the Care and Use of Laboratory Animals published by the US National Institutes of Health (Publication, 8^th^ Edition, 2011). Healthy male C57BL/6 mice (20‐25 g, 8 weeks old) were purchased from WeiTongLiHua Company, Beijing, China, and housed with free access to standard rodent chow and water.

### 2.3 Experimental settings

A total of 45 mice were randomly divided into three groups, including Sham, Doxorubicin and Doxorubicin + Ursolic acid treatment groups (n = 15 per group). Doxorubicin was dissolved in the vehicle DMSO (2%). In the Doxorubicin + Ursolic acid group, daily subcutaneous injections of ursolic acid in 100 μL DMSO (80 mg/kg/day) were performed for a week before intraperitoneal administration of doxorubicin (15 mg/kg) in 200 μL double distilled water (DDW)^21^; in the Doxorubicin group, daily subcutaneous injections of DMSO were performed for a week before single intraperitoneal administration of doxorubicin in 100 μL DDW (15 mg/kg). The Sham group was administered equivalent volumes of DMSO (daily for a week) and DDW subcutaneously and intraperitoneally respectively. Then, 7 and 28 days were selected as time‐points for early and late heart injury due to doxorubicin respectively.^6^ Echocardiography was performed to evaluate cardiac function immediately before doxorubicin treatment and at 7 and 28 days thereafter. To assess the effects of ursolic acid on early injury to the heart, 10 mice in each group were killed at 7 days, obtaining heart specimens. Of these, five hearts were used for NO and ROS detection, while the remaining five were employed in TUNEL staining and Western blot for apoptosis‐related proteins. To evaluate the effects of ursolic acid on late injury, the remaining five mice in each group were killed at 28 days.

### 2.4 Echocardiography

Echocardiography was performed to measure cardiac function on a Prospect High Resolution Imaging System (S‐Sharp Corporation, Taiwan, China) as previously reported.^6^ In brief, after anaesthetized with inhalation of 1.0% isoflurane, the mice were placed in a supping position on the platform with heating to maintain the body temperature. The heart was imaged in the 2‐D mode in the parasternal short‐axis view with a depth setting of 2 cm. The M‐mode cursor (40 MHz) was positioned perpendicular to interventricular septum and posterior wall of left ventricle at the level of papillary muscles from the 2‐D mode. The sweep speed was 100 mm/s for the M‐mode. Left ventricular end diastolic dimension and left ventricular systolic dimension were measured. LVEF and LVFS were calculated and selected as the indexes of the cardiac function. The value of LVEF and LVFS averaged from at least three separate cardiac cycles.

### 2.5 Assessment of cardiac NO production

Cardiac NO production was quantitated by evaluating its oxidation products (nitrate and nitrite) using the nitrate reductase method,^22^ in which nitrate is reduced to nitrite by nitrate reductase; nitrite levels were measured by the Griess reaction. A total of five mice in each group were killed 1 week after doxorubicin treatment. Each heart extracted was cut into two pieces, which were snap frozen. The first half was homogenized in precooled normal saline; after centrifugation of the homogenate at 4°C and 2000 r/min for 15 minutes, the supernatant was assessed with an NO Assay Kit (Nanjing Jiancheng Bioengineering Corporation, China, A012) according to the manufacturer's instructions. Absorbance was measured spectrophotometrically at 530 nm. Total NO content (μmol/g protein) was determined using a standard curve, and each sample was tested in triplicate.

### 2.6 Measurement of reactive oxygen species production

DCFH‐DA method was used to assess ROS levels in myocardium specimens.^23^ The other half of snap frozen myocardium from mice killed at 1 week after doxorubicin treatment was serially sectioned at 4 μm thickness and incubated with 2,7‐dichlorofluorescin diacetate (DCFH‐DA) (20 μmol L^−1^) (ROS assay kit, Nanjing Jiancheng Bioengineering Corporation, China, E004) at 37°C for 60 minutes in the dark. Five random high power fields from each tissue section were observed under an Olympus IX71 fluorescence microscope (Tokyo, Japan). Fluorescence intensities of the sections stained for ROS were determined with the Image‐Pro Plus software.

### 2.7 Creatine kinase MB (CKMB) measurement

The CKMB measurement was done as previously reported.^6^ In brief, five mice in each group were killed on day 7 after doxorubicin treatment, and the blood samples were withdrawn immediately through the right atrium. After standing at room temperature for 30 minutes, the samples were centrifuged (634 *g*, 10 minute), and serum samples were collected. CKMB were evaluated by a full‐automatic biochemical detection machine (COBAS C 311, Roche Diagnostics GmbH, Germany) using specific CKMB detection kits (BIOBASE, Jinan, Shandong, China). The procedure was performed following manufacture's manual.

### 2.8 Terminal dUTP nick end‐labelling (TUNEL) assay

Heart samples were fixed with 10% formaldehyde, paraffin embedded and cut into 4 μm thick sections. In Situ Cell Death Detection Kit (TUNEL fluorescence FITC kit; Roche, Indianapolis, USA) was used to assess apoptosis in mouse heart samples. After three washes with PBS, the samples were fixed with 4% paraformaldehyde for 1 hour, and permeabilized in 0.1% Triton X‐100 sodium citrate buffer for 2 minutes. Then, sections were randomly selected for TUNEL staining and visualized with a DAB kit. Nuclei were counterstained with haematoxylin. All and TUNEL‐positive nuclei were counted in five randomly selected fields of view per tissue section in a blinded manner; the results were expressed as the number of TUNEL‐positive nuclei per field.

### 2.9 Western blot

Snap frozen heart samples obtained at 7 days after treatment with doxorubicin were washed twice with cold PBS and resuspended in cold lysis buffer containing 20 mmol/L HEPES (pH 7.5), 150 mmol/L NaCl, 1 mmol/L EDTA, 0.5% Triton X‐100 and protease inhibitors (Roche). Equal amounts of total protein (40 μg) were separated by SDS‐PAGE, transferred onto nitrocellulose membranes and blocked for 1 h in blocking solution at room temperature. To assess protein expression, the membranes were incubated overnight at 4°C with primary antibodies, followed by treatment with antimouse or anti‐rabbit secondary antibodies conjugated to horseradish peroxidase (Zymed, Inc., South San Francisco, CA) for 1 hour at room temperature. An enhanced chemiluminescence ECL Plus system (Amersham Biosciences UK, Little Chalfont, UK) was used for visualization. β‐actin was used as an internal control. eNOS monomer and dimer were detected by low‐temperature SDS‐PAGE as the previously reported procedures with some modification .^24^ Briefly, total proteins were incubated in 1 × Laemmli buffer without 2‐mercaptoethanol at 37°C for 5 minutes. The samples were then resolved on 4%‐12% Bis‐Tris gradient gels (Bio‐Rad). Gels and buffers were equilibrated at 4°C before electrophoresis, and the buffer tank was placed in a 4°C cold room during electrophoresis. Then, gels were transferred to PVDF membranes as a routine Western blot.

### 2.10 Haematoxylin and eosin (H&E) and Masson's trichrome staining

Five mice in each group were killed at 28 days after doxorubicin treatment, and heart samples were harvested, paraffin embedded and cut into 4 μm thick sections. Then, the sections were submitted to haematoxylin and eosin or Masson's trichrome staining for histological analysis and collagen level assessment respectively. Interstitial fibrotic areas were evaluated with an image analysis software (Image‐pro plus 6.0; Media Cybernetics LP, Washington, USA). Collagen volume fraction (CVF) was derived as the ratio of collagen to total area.

### 2.11 Statistical analysis

Data are mean ± standard deviation (SD). One‐way anova followed by Bonferroni/Dunn tests was used for multiple‐group comparisons. *P *<* *0.05 was considered statistically significant.

## 3 RESULTS

### 3.1 Ursolic acid preserves cardiac function in mice treated with doxorubicin

**Figure 1:**
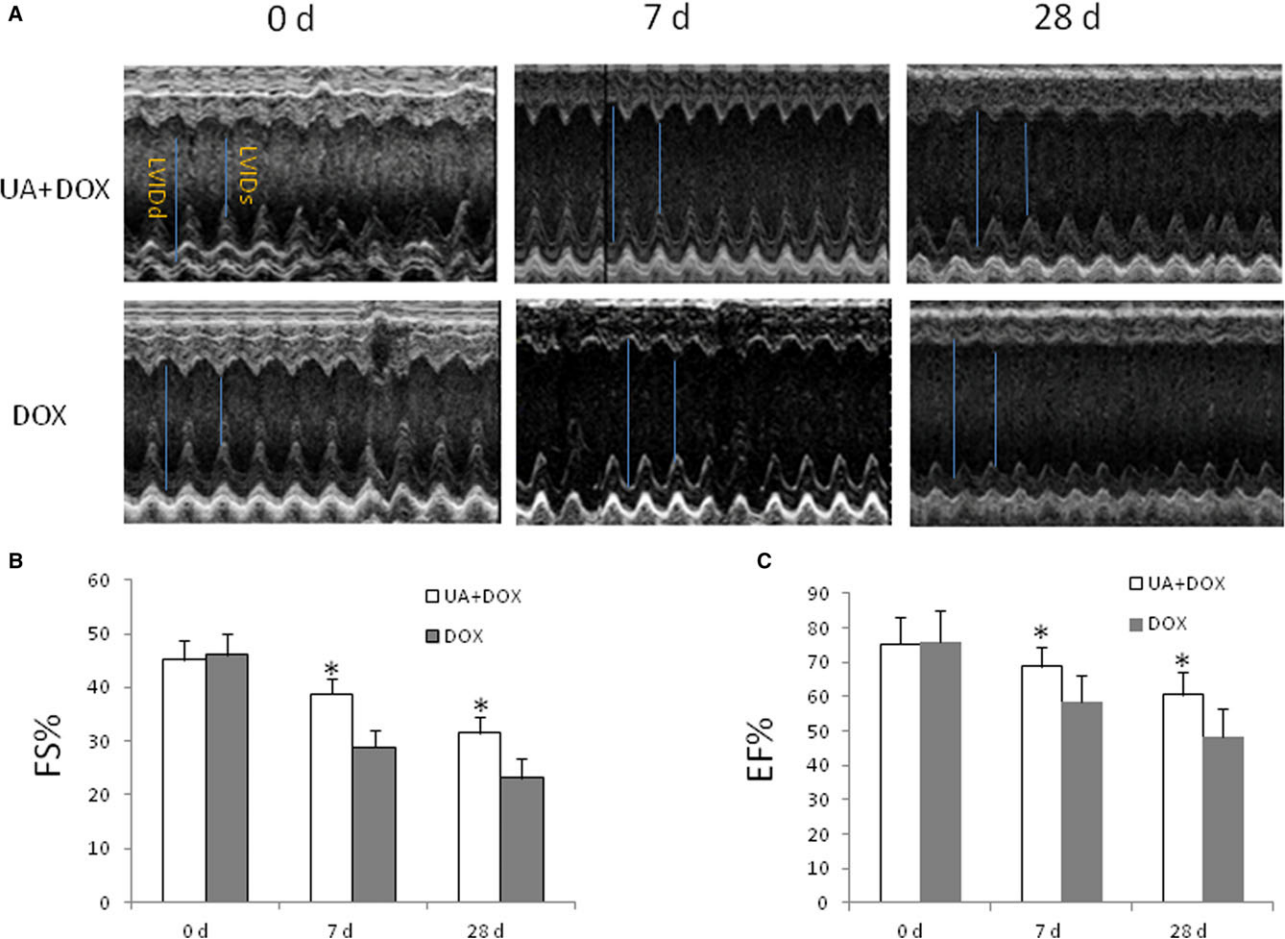
Ursolic acid preserves cardiac function in mice treated with doxorubicin in both early and late injury phases. A, Heart echocardiography. Upper panel, Doxorubicin + Ursolic acid group (UA + DOX); lower panel, Doxorubicin group (DOX). LVIDd, left ventricle internal diameter in diastole; LVIDs, left ventricle internal diameter in systole. B, Ursolic acid treatment significantly improved FS at both 7 and 28 days, compared with the Doxorubicin control group (n = 5, *P < 0.05). C, Ursolic acid significantly improved EF at both 7 and 28 days, compared with the doxorubicin control (n = 5, *P < 0.05)

First, echocardiography was used to assess the effects of ursolic acid on cardiac function in mice treated with doxorubicin in both early (7th day) and late (28th day) injury phases. We found that at both 7 and 28 days, administration of ursolic acid significantly improved FS and EF, compared with the control group (Figure 1). This indicated ursolic acid preserved cardiac function in mice treated with doxorubicin in both early and late injury phases.

### 3.2 Ursolic acid increases NO production and decreases ROS levels in the heart after doxorubicin treatment

**Figure 2:**
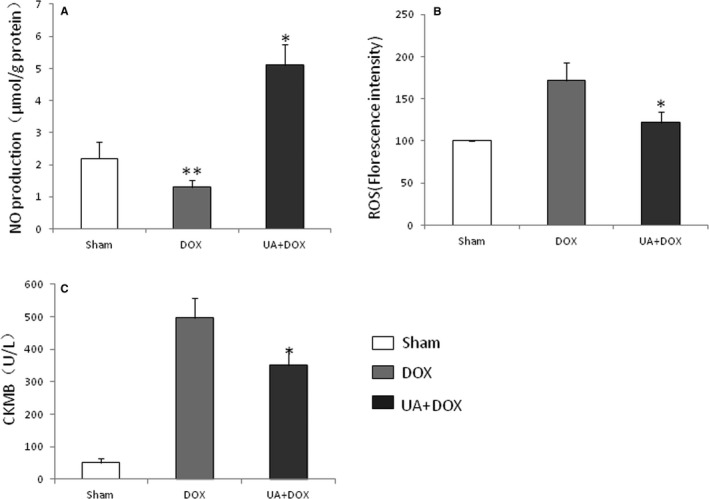
Ursolic acid increases NO production, inhibits ROS generation and decreases CKMB releasing in the mouse heart after doxorubicin treatment. Mice were divided into three groups: Sham group, Doxorubicin group and Doxorubicin + Ursolic acid group. A, DOX significantly decreased NO production compared with the sham (n = 5, **P < 0.05), while ursolic acid significantly increased NO production compared with the doxorubicin control (n = 5, *P < 0.01). B, Ursolic acid significantly inhibited ROS production compared with doxorubicin (n = 5, *P < 0.05). C, Ursolic acid significantly decreased CKMB releasing from the hearts in mice compared with doxorubicin (n = 5, *P < 0.05)

To evaluate the effects of ursolic acid on NO and ROS production in the heart after treatment with doxorubicin, mice in each group were killed and heart specimens were harvested at 7 days after doxorubicin treatment. The results showed that ursolic acid significantly increased NO production and decreased ROS levels in the heart after doxorubicin treatment, compared with the control group (Figure 2A and B).

### 3.3 Ursolic acid decreases CKMB levels in the heart after doxorubicin treatment

To evaluate the effects of ursolic acid on CKMB production in the heart after treatment with doxorubicin, mice in each group were killed and heart specimens were harvested at 7 days after doxorubicin treatment. The results showed that ursolic acid significantly decreased CKMB level in the heart after doxorubicin treatment, compared with the control group (Figure 2C).

### 3.4 Ursolic acid decreases apoptosis of cardiac cells in mice treated with doxorubicin

**Figure 3:**
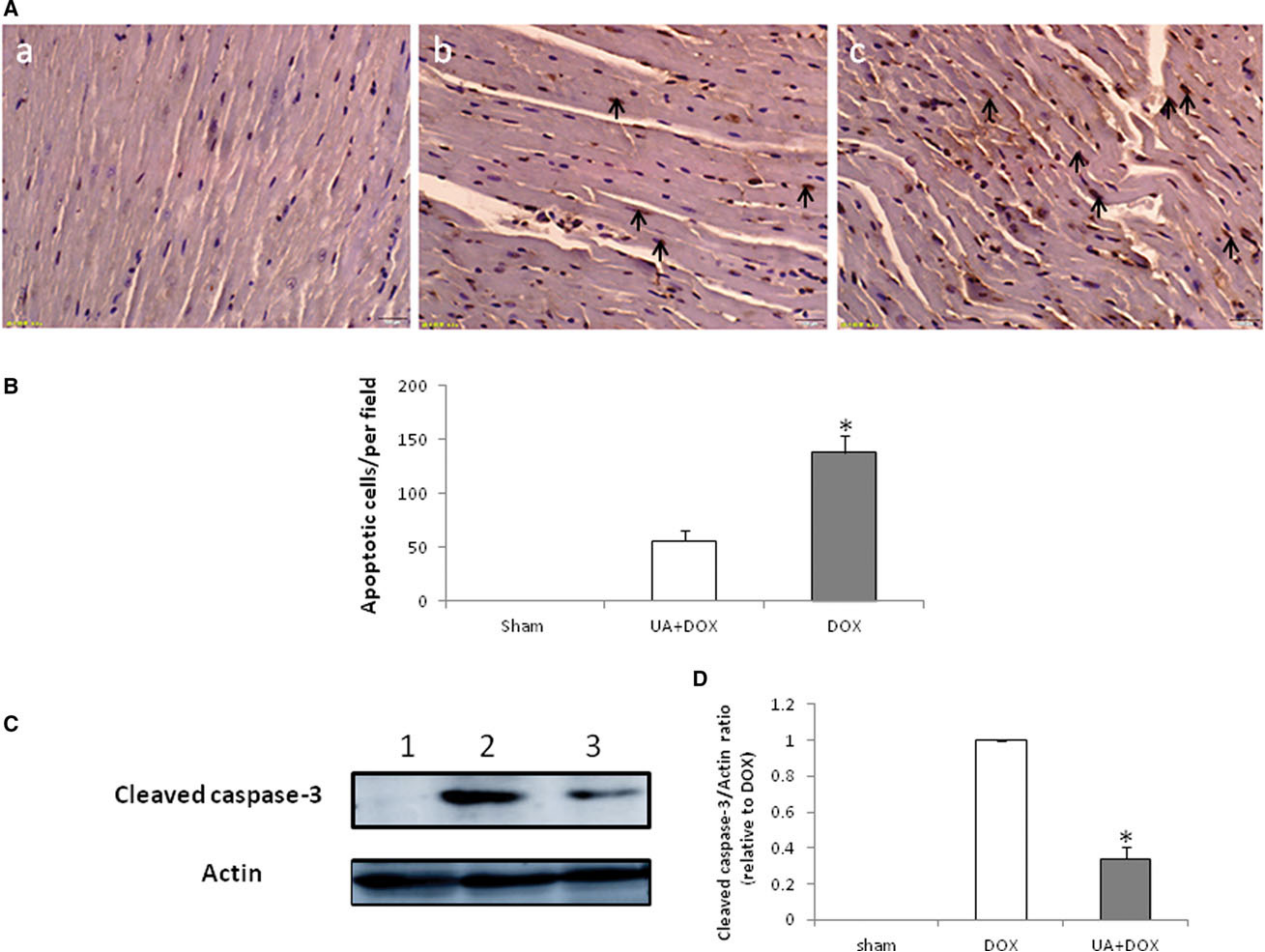
Ursolic acid decreases apoptosis in the heart of mice treated with doxorubicin. A, TUNEL staining of heart samples. a, Sham group; b, Doxorubicin + Ursolic acid group; c, Doxorubicin group (Bar = 20 μm). B, Ursolic acid significantly decreased cardiac cell apoptosis in mice treated with doxorubicin compared with the doxorubicin control (n = 5, *P < 0.01). Apoptotic cell nuclei were stained brown (arrow). C, Western blot detection of cleaved caspase‐3 in the mouse heart after treatment with doxorubicin. Lanes 1 to 3 represent the Sham, Doxorubicin and Doxorubicin + Ursolic acid treatment groups respectively. E. Ursolic acid significantly decreased cleaved caspase‐3 levels in the hearts of mice compared with doxorubicin (n = 5, *P < 0.01)

To assess the effects of ursolic acid on cardiac cell apoptosis in mice treated with doxorubicin in the early injury phase, mice in each group were killed 1 week after ursolic acid treatment, and heart specimens were harvested for TUNEL staining. The results showed that ursolic acid significantly decreased cardiac cell apoptosis in mice treated with doxorubicin, compared with the control group (Figure 3A and B). In addition, ursolic acid reduced cleaved caspase‐3 levels in the mouse heart after doxorubicin treatment, compared with controls (Figure 3C). These findings indicated that ursolic acid preserved cardiac function in mice treated with doxorubicin by reducing cardiac cell apoptosis.

### 3.5 Ursolic acid inhibits inflammation and fibrosis in the mouse heart after treatment with doxorubicin

**Figure 4:**
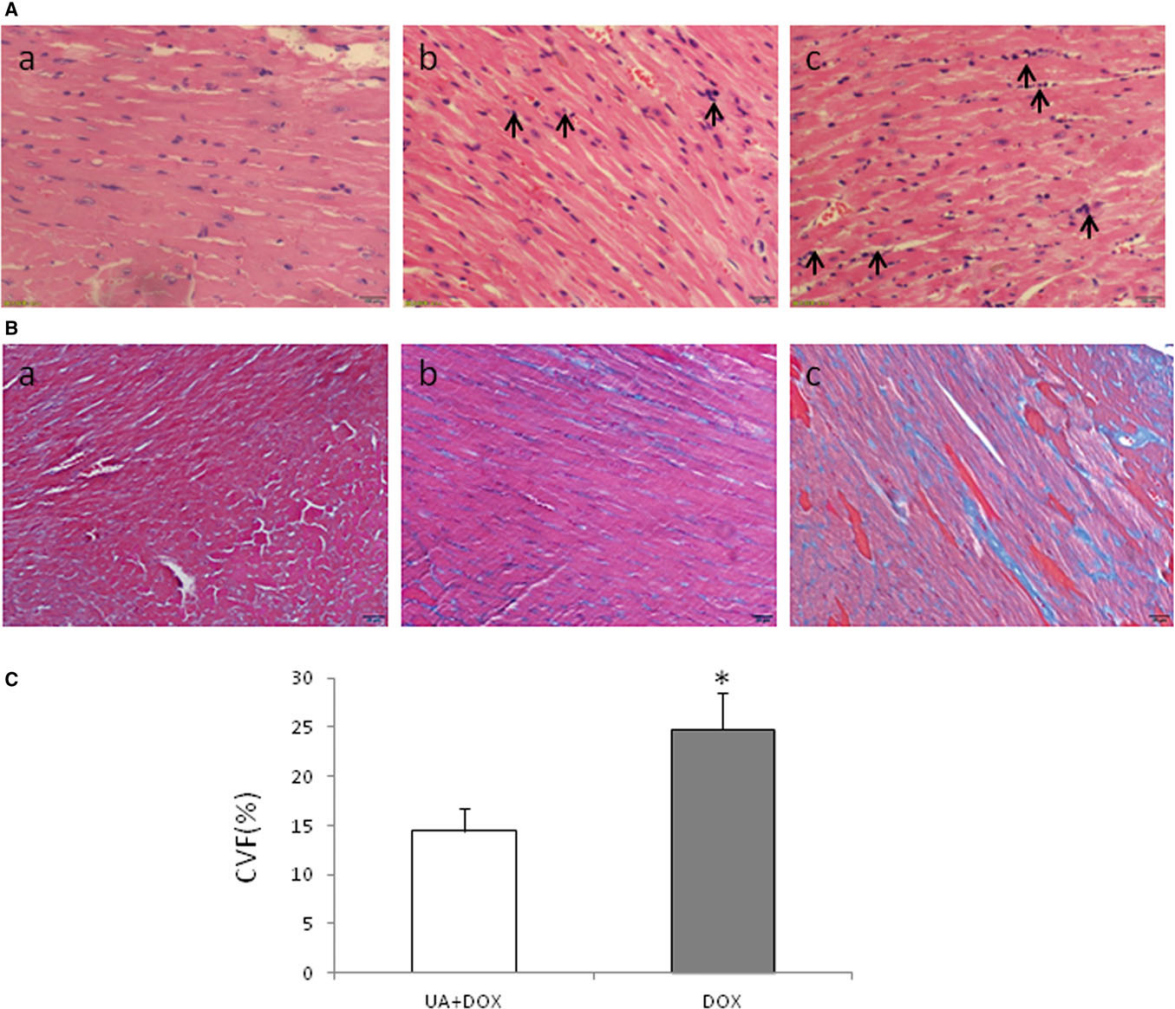
Ursolic acid inhibits inflammation and fibrosis in the mouse heart after doxorubicin treatment. A, H&E staining of heart. a, Sham group; b, Doxorubicin + Ursolic acid group; c, Doxorubicin group. Ursolic acid overtly decreased inflammatory cell infiltration compared with doxorubicin (Bar = 50 μm; arrows indicate inflammatory cells). B, Masson's staining of heart samples. a, Sham group; b, Doxorubicin + Ursolic acid group; c, Doxorubicin group (Bar = 20 μm). Ursolic acid significantly decreased fibrosis compared with the doxorubicin control (n = 5, *P < 0.01)

To further evaluate the effects of ursolic acid on the heart in the late injury phase, mice were killed at 4 weeks, and heart samples were obtained and paraffin embedded. The H&E and Masson's staining methods were performed. The results showed that ursolic acid significantly inhibited inflammatory cell infiltration into the heart (Figure 4A) as well as cardiac tissue fibrosis (Figure 4B and C) in mice treated with doxorubicin.

### 3.6 Ursolic acid increases AKT and eNOS phosphorylation levels in the heart of mice treated with doxorubicin

**Figure 5:**
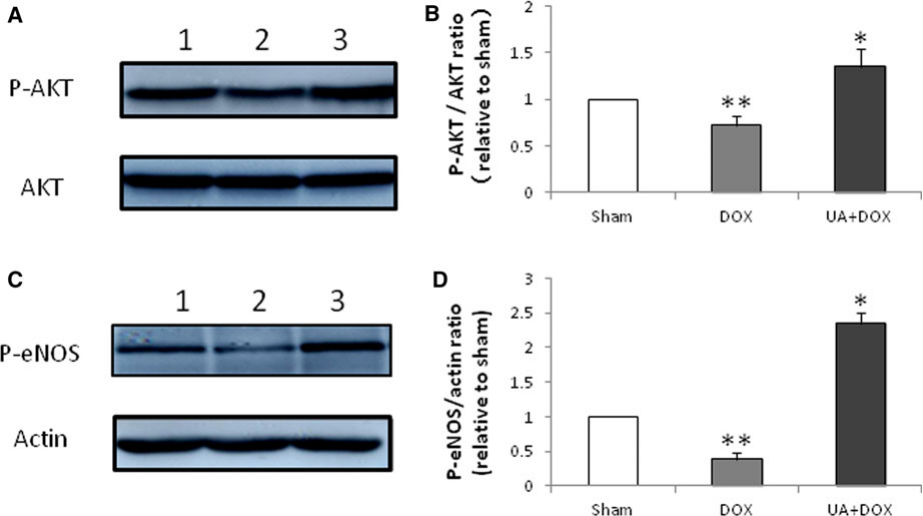
Effects of ursolic acid on phosphorylation levels of AKT and eNOS. Protein levels were detected by immunoblot. A, Effects of ursolic acid on AKT and p‐AKT. Lanes 1 to 3 represent the Sham, Doxorubicin and Doxorubicin + Ursolic acid treatment groups respectively. B, doxorubicin significantly decreased AKT phosphorylation compared with sham (n = 5, **P < 0.05), while ursolic acid significantly increased AKT phosphorylation compared with doxorubicin (n = 5, *P < 0.05). C, Effect of ursolic acid on p‐eNOS. Lanes 1 to 3 represent the Sham, Doxorubicin and Doxorubicin + Ursolic acid treatment groups respectively. D, doxorubicin significantly decreased eNOS phosphorylation compared with sham (n = 5, **P < 0.01), while ursolic acid significantly increased eNOS phosphorylation compared with doxorubicin (n = 5, *P < 0.01)

AKT and eNOS play important roles in cell apoptosis. Therefore, we performed Western blot detection of AKT and eNOS in the mouse heart harvested 1 week after doxorubicin administration. The results showed that ursolic acid significantly increased the phosphorylation levels of AKT and eNOS in the mouse heart after doxorubicin treatment (Figure 5A‐D), which indicated that the AKT‐eNOS pathway played an important role in the anti‐apoptotic effects of ursolic acid in cardiac cells.

### 3.7 Ursolic acid down‐regulates NOX4 and inhibits eNOS uncoupling in the mouse heart after doxorubicin treatment

**Figure 6:**
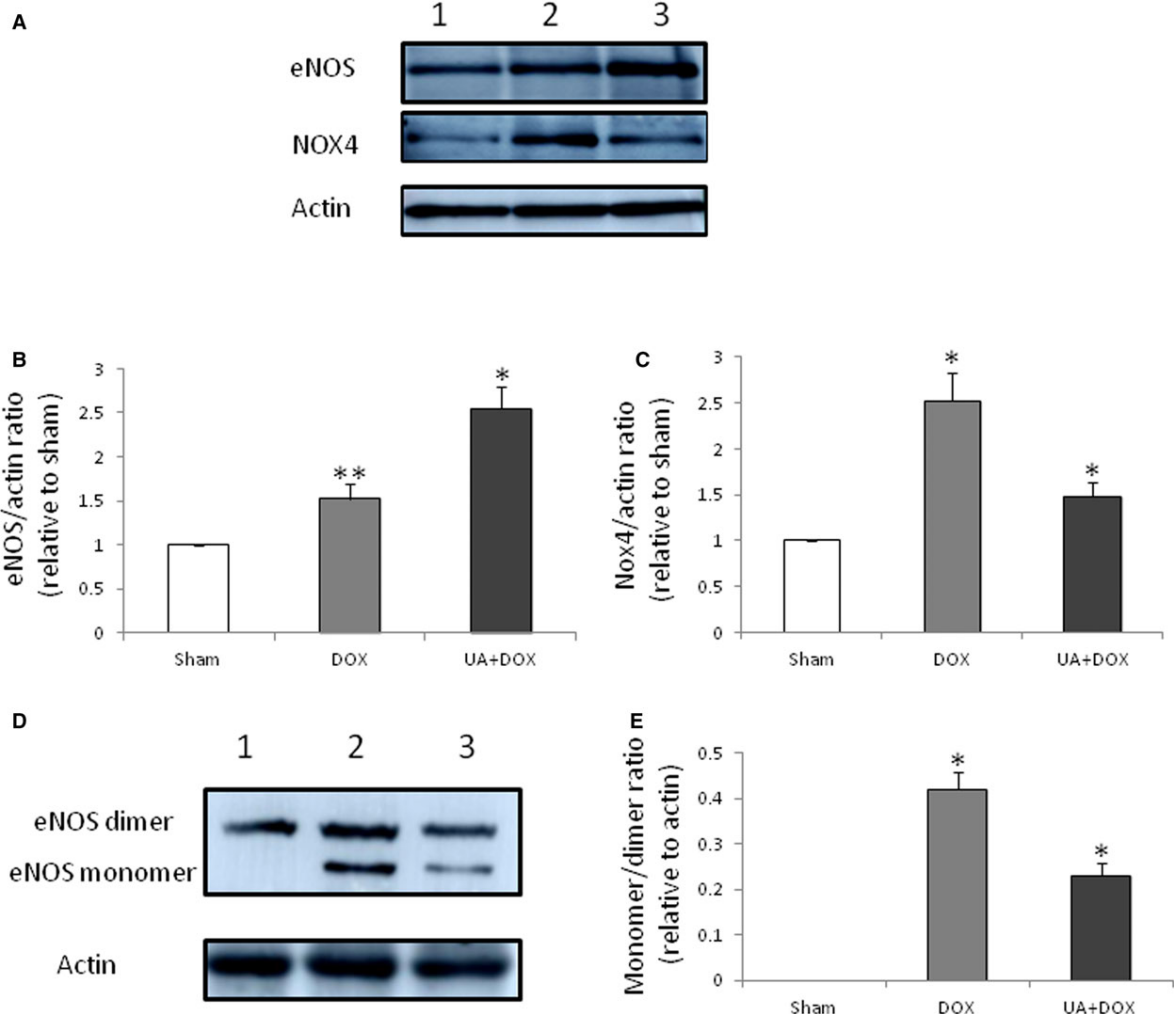
Effects of ursolic acid on eNOS and NOX4. A, Effect of ursolic acid on eNOS. Lanes 1 to 3 represent the Sham, Doxorubicin and Doxorubicin+Ursolic acid treatment groups respectively. B, doxorubicin significantly increased eNOS expression compared with sham (n = 5, **P < 0.05), while ursolic acid significantly increased eNOS expression compared with doxorubicin (n = 5, *P < 0.01). C, doxorubicin significantly increased NOX4 expression compared with sham (n = 5, *P < 0.01), while ursolic acid significantly decreased Nox4 expression compared with doxorubicin (n = 5, *P < 0.01). D, Effect of ursolic acid on the expression of eNOS monomer and dimer. Lanes 1 to 3 represent the Sham, Doxorubicin and Doxorubicin + Ursolic acid treatment groups respectively. E. doxorubicin significantly increased eNOS monomer/dimer ratio compared with sham (n = 3, *P < 0.05), while ursolic acid significantly decreased eNOS monomer/dimer ratio compared with doxorubicin (n = 3, *P < 0.05)

Western blot showed that ursolic acid increased eNOS levels, which were also enhanced by doxorubicin (Figure 6A and B). In addition, doxorubicin up‐regulated NOX4 (Figure 6A and C), an important factor in eNOS uncoupling.^25^ To assess the effect of ursolic acid on eNOS uncoupling, the levels of NOX4, eNOS dimer and monomer were evaluated 1 week after ursolic acid administration. The results showed that ursolic acid significantly reduced NOX4 expression (Figure 6A and C) and decreased eNOS monomer/dimer ratio (Figure 6D and E) in the mouse heart after treatment with doxorubicin, indicating that ursolic acid inhibited eNOS uncoupling although it up‐regulated eNOS in the mouse heart after doxorubicin treatment (Figure 6A and C).

## 4 DISCUSSION

The mechanisms of doxorubicin‐associated cardiac toxicity are not fully elucidated; however, cardiac cell apoptosis by ROS is a known side effect of doxorubicin. Multiple reports have demonstrated that doxorubicin‐induced cardiac toxicity is decreased by anti‐apoptotic methods in various animal models.^26^, ^27^, ^28^ It is known that ursolic acid protects the heart from ischaemic reperfusion injury or infarction by anti‐apoptotic activities.^29^, ^30^, ^31^ Therefore, via anti‐apoptosis, ursolic acid may decrease the side effects of doxorubicin on the heart. In this study, ursolic acid decreased apoptosis of cardiac cells, and significantly improved cardiac function in mice treated by doxorubicin. We further demonstrated that ursolic acid increased expression of eNOS and phosphorylation of AKT and eNOS while decreasing ROS amounts. Increased expression and phosphorylation levels of eNOS by ursolic acid, with subsequently increased NO production, is an important downstream mechanism in the survival signalling pathway of endothelial cells.^25^, ^32^, ^33^ Thus, activation of AKT and eNOS (expression and phosphorylation) is one of the mechanisms by which ursolic acid prevents doxorubicin‐related cardiac toxicity.

However, Kalivendi et al^34^ demonstrated that doxorubicin treatment also increases eNOS transcription and protein levels in bovine aortic endothelial cells, and pre‐treatment with antisense eNOS mRNA causes a decrease in doxorubicin‐induced apoptosis. Evidence suggests that eNOS‐dependent ROS formation plays a role in doxorubicin‐induced myocardial dysfunction. Den et al^35^ found that genetic disruption of eNOS transcription protects against doxorubicin‐associated cardiac dysfunction. Meanwhile, overexpression of eNOS exacerbates the pathological response to doxorubicin in the heart, and doxorubicin‐induced levels of cardiac ROS synthesis are highest in eNOS‐transgenic mice and least in knockout counterparts.^25^ As shown above, doxorubicin increased eNOS expression, which was further enhanced by ursolic acid. However, cardiac dysfunction was alleviated even with eNOS up‐regulation. This might be explained by the fact that ursolic acid affects eNOS uncoupling.

The process of eNOS uncoupling plays an important role in ROS production. Stimulated by pathologic factors, NADPH oxidases are up‐regulated and generate superoxide (O2‐·), which is converted by SOD into H_2_O_2_, increasing eNOS expression and subsequently NO levels. O2‐· and NO rapidly bind to form peroxynitrite (ONOO‐), which oxidizes the essential cofactor of eNOS BH4 to BH3 and BH2. Consequently, oxygen reduction by eNOS is uncoupled from NO formation, and functional eNOS is converted into a dysfunctional O2‐·‐generating enzyme, which contributes to oxidative stress.^36^ Thus, increasing eNOS protein levels without a concomitant elevation of endothelial BH4 levels may lead to eNOS uncoupling and subsequent enhanced oxidative stress; increasing eNOS protein levels is only beneficial with guaranteed eNOS functionality. As shown above, ursolic acid increased eNOS expression, decreased NOX4 levels and eNOS monomer/dimer ratio in the heart. By reducing NOX4 amounts, ursolic acid inhibited eNOS uncoupling, which resulted in elevated NO and reduced ROS levels in the heart after doxorubicin treatment. This is in line with findings by Steinkamp‐Fenske K et al that ursolic acid from the Chinese herb Danshen (*Salvia miltiorrhiza* L.) up‐regulates eNOS and reduces NAPDH oxidase‐related Nox4 expression in human endothelial cells.^25^ Therefore, inhibiting eNOS uncoupling induced by doxorubicin constitutes an important mechanism by which ursolic acid protects the heart from doxorubicin‐related injury.

**Figure 7:**
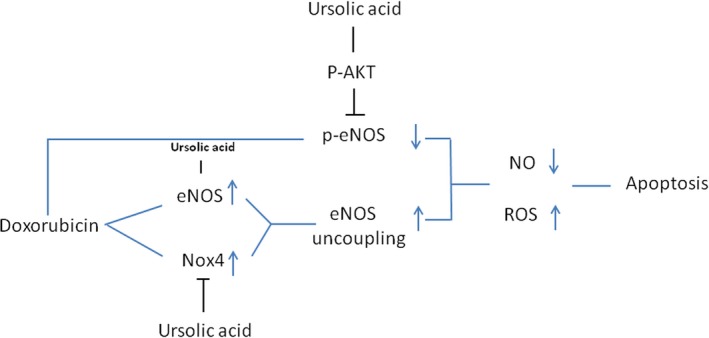
Schematic representation of study outcome. Doxorubicin increased eNOS and NOX4 levels, which results in eNOS uncoupling and decreased eNOS phosphorylation, enhancing ROS production. Meanwhile, ursolic acid increased eNOS production and phosphorylation, and inhibited NOX4 levels, decreasing ROS levels through eNOS activation and reduced eNOS uncoupling

In summary, ursolic acid preserves cardiac function and decreases cardiac cell apoptosis after doxorubicin treatment in mice. Mechanistically, ursolic acid increases the phosphorylation levels of AKT and eNOS, and inhibits eNOS uncoupling induced by doxorubicin; this results in increased NO levels and decreased ROS production, preventing cardiac cell apoptosis associated with doxorubicin toxicity (Figure 7). These findings suggest that ursolic acid may constitute a potential molecule for the prevention of doxorubicin‐induced cardiac toxicity in clinical practice.

## CONFLICT OF INTEREST

The authors declare no conflict of interest.
